# Association between the systemic immune‐inflammation index and erectile dysfunction: A cross‐sectional study

**DOI:** 10.1002/iid3.1363

**Published:** 2024-08-02

**Authors:** Di Chen, Fuchang Chen, Quanhai Luo, Wenji Fan, Changsheng Chen, Gang Liu

**Affiliations:** ^1^ The Department of Urology Reproductive Hospital of Guangxi Zhuang Autonomous Region Nanning China; ^2^ Graduate School Guangxi Medical University Nanning China; ^3^ The Second Department of Urology The First People's Hospital of Qinzhou City Qinzhou China; ^4^ The Department of Urology The Second People's Hospital of Nanning City Nanning China; ^5^ The Department of Urology People's Hospital of Guangxi Zhuang Autonomous Region Nanning China

**Keywords:** erectile dysfunction, lymphocyte, national health and nutrition examination survey, neutrophil, platelet, systemic immune‐inflammation index

## Abstract

**Background:**

Erectile dysfunction (ED) is associated with inflammation. The systematic immune‐inflammation index (SII), as a new inflammation marker, was applied to predict the risk of diseases. However, no research explores the relationship between SII and ED. Hence, the purpose of this study was to investigate the association between SII and ED.

**Methods:**

Related data were obtained from the National Health and Nutrition Examination Survey (NHANES) 2001−2004. Based on self‐report, all participants were classified into ED and non‐ED group. Weighted multivariate regression analysis the relationship between categorical SII and ED in unadjusted and adjusted models. Restricted cubic spline (RCS) was used to examine the association of continuous SII and ED risk. Furthermore, the association between categorical SII and the risk of ED was evaluated among subgroups of age, body mass index, hypertension, diabetes and cardiovascular disease. Finally, weighted multivariate regression analysis and RCS were performed to assessed the connection between SII and the risk of severe ED.

**Results:**

Initially, data on 21,161 participants were obtained. After implementing the inclusion and exclusion criteria, 3436 participants were included in analyses. Weighted multivariate regression analysis demonstrated that Q4 group SII was associated with an increased risk of ED (OR = 1.03, 95% confidence intervals: 1.00−1.05, *p* = .03). RCS showed SII was nonlinearly associated with the risk of ED, and the inflection point of SII was at 485.530. In addition, subgroup analyses demonstrated that participants in the SII > 485.530 group had a higher ED risk than SII ≤ 485.530 group among subgroups of age ≥50, hypertension, and non‐diabetes. Weighted multivariate regression analysis and RCS found no relationship of SII and the risk of severe ED.

**Conclusion:**

In US adults, SII > 485.530 was correlated with an increased risk of ED. While, no significant association between SII and severe ED risk. Additional studies are required to support our results.

## Graphical abstract

This is the first cross‐sectional study that describes the association between systemic immune‐inflammation index (SII) and erectile dysfunction (ED). our study found a positive result that SII > 485.530 can significantly increase the risk of ED.

## 1 INTRODUCTION

Erectile dysfunction (ED) is a common male sexual dysfunction disease, and is defined as the persistent inability to attain or maintain a penile erection sufficient for satisfactory sexual performance.^1^ The prevalence of ED in the general population is 19−52%.^2^ In US, Holland, and Brazil, the new ED patients were 19−66 per 1000 men per year.^3^, ^4^, ^5^ However, due to differences in geography, culture, ethnicity, society, and economy, the incidence of ED in different regions varies widely. As a multifactorial disease, ED is affected by cavernosa, hormonal, vascular, neurogenic, and drug‐induced. Advanced age, obesity, smoking, depression, hypertension, obstructive sleep apnoea, and diabetes have been described as risk factors for the development of ED.^6^ Available evidence suggests systemic inflammatory conditions lead to endothelial dysfunction, which is associated with several vascular disease.^7^ Duo to the smaller size and poor tolerance of penile arteries, ED may be an early indicator of systemic endothelial dysfunction.^8^ Thus, much research attention has focused on the relationship between inflammation and ED.

Inflammation is a physiological response that protects organisms from infections or tissue injury. However, the imbalance of immune response and inflammation leads to various diseases. Several immune‐inflammatory markers, such as lymphocytes, neutrophils, neutrophil‐to‐lymphocyte ratio (NLR), platelet‐to‐lymphocyte ratio (PLR), and C‐reactive protein (CRP) levels, commonly used to evaluated the status of system inflammation and have been reported to be associated with the development of ED. Liu et al. performed a meta‐analysis including 12 studies and found a higher CRP levels in ED patients compared to healthy controls. CRP attenuates vasodilation by inhibiting nitric oxide synthase activity in the vascular endothelium and increasing the expression of vascular cell adhesion molecule‐1. Meanwhile, they found PDE5I therapy can improve the abnormal CRP levels.^9^ Sembel et al. performed a compare study including 101 ED patients and found a higher PLR, NLR and CRP than sexually active male.^2^ Systemic immune‐inflammation index (SII), as a new integrated indicator based on peripheral blood lymphocytes, neutrophils, and platelets, was reported a relationship with hyperlipoidemia, cardiogenic shock, hypertension, and depression.^10^, ^11^, ^12^, ^13^ However, to data, no research has reported the correlation between SII and ED.

In this study, we explore the association between SII and the risk of ED based on the National Health and Nutrition Examination Survey (NHANES), and found a cutoff of SII in ED risk.

## 2 MATERIALS AND METHODS

### 2.1 Data extraction and screening

The NHANES database contains several data modules: demographics, dietary, examination, laboratory, and questionnaire. The National Center for Health Statistics Research Ethics Review Board approved the NHANES, and participants provided written consent. All detailed NHANES study designs and data are publicly available at www.cdc.gov/nchs/nhanes. After inclusion, participants receive a unique code, which is used to position in each module. Furthermore, each item will be assigned a unique number. Even in different cycles of NHANES, the same item has the same number. Therefore, data from different cycles can be pooled to increase sample size.

Because ED related questionnaire was only available for participants 20 years and older from 2001 to 2004, we pooled data from 2001 to 2002 and 2003−2004 NHANES cycles. Demographic data included age, race, education level, annual household income, and marital status. Body mass index was obtained from the examination data. The complete blood cell count was obtained from the laboratory data. Information on smoking, alcohol consumption, physical activity, diabetes, hypertension, cardiovascular disease, and ED were obtained from the questionnaire module. The inclusion criteria were as follows: (1) ED data based on questionnaire KIQ400 was “Always or almost always able,” “Usually able,” “Sometimes able,” and “Never able”; (2) data on neutrophil, lymphocyte and platelet were nonnull value. The exclusion criteria: (1) data on education level, marital status, annual household income, smoking, alcohol consumption, and physical activity were “unknown,” “null,” and “refuse to answer”; (2) data on hypertension, diabetes, and cardiovascular disease were “unknown” and “null”; (3) participants who diagnosed with prostate cancer.

### 2.2 Outcome and exposure variables

The exposure variable was SII, which was calculated using the following formula: SII = platelet count × neutrophil count ÷ lymphocyte count.^14^

The code number of lymphocytes, neutrophils, and platelets are “LBDLYMNO,” “LBDNENO,” and “LBXPLTSI,” respectively. Venous blood samples were collected at the mobile examination center (MEC). The Beckman Coulter MAXM were used to detect blood cell count in 2001−2004.

The outcome variable was whether the participant had ED. Questionnaire data module contains prostate condition item recorded related questionnaire. Participants need to answer the question “Ability to maintain an erection?”. If participants responded “Always or almost always able” and “Usually able,” they were categorized as the non‐ED group. Otherwise, they were placed in the ED group. Of these, participants who responded “Never able” were categorized as the severe ED group.

## 3 COVARIATES

Besides outcome and exposure variables, the remaining variables were covariates. Some of these covariates were treated as categorical variables. Age was categorized into <50, and 50 years of age or older. Annual family income was categorized into <$20,000 and ≥$20,000.

Body mass index was categorized into <25, 25−29.99, and ≥30 kg/m^2^. Some covariates were defined. Education level was categorized into less than high school, high school, and college or above. Marital status recoded into two groups: married or living with partner and living alone. Smoking and drinking both were classified as yes and no. History of diabetes, hypertension, and cardiovascular disease were also classified as yes and no. Of note, cardiovascular disease included coronary heart disease, angina, and stroke. Other covariates were categorized based on NHANES categorization.

## 4 STATISTICAL ANALYSIS

Analyses used survey methods for complex sampling designs with appropriate strata, primary sampling units, and sampling weights. Because some variables were measured at the MEC, the MEC examination weights recorded as “WTMEC2YR” were used for all analyses. Continuous variables were expressed as means and 95% confidence intervals (CI), whereas categorical variables represented as the number of cases (percentage). The Wilcoxon rank‐sum test for complex survey samples and the chi‐square test were used to compare two groups. SII was divided into four same‐group distances: 25% (Quartile 1, Q1), 50% (Quartile 2, Q2), 75% (Quartile 3, Q3), and 100% (Quartile 4, Q4). The Q1 SII was the lowest level and the highest in Q4.

Weight multivariable logistic regression was used to analyze the association between SII and the risk of ED in three models. Molde 1: No covariates were adjusted. Mode 2: age, race, education level, marital status, annual family income, and body mass index were adjusted. Model 3: All covariates were adjusted. Additionally, restricted cubic spline (RCS) was used to explore the potential association between continuous SII and the odd ratio of ED and calculate the inflection point of SII. Subgroup analysis stratified by age, body mass index, hypertension, diabetes, and cardiovascular disease was applied to examine the association between SII and ED risk in model 3. The interaction effect of the SII‐ED relation and these subgroups were assessed. Finally, weight multivariable logistic regression and RCS evaluated the relationship between SII and severe ED. Two‐tailed *p* Values less than .05 were considered statistically significant. Data analysis was performed with R software version 4.2.2 (http://www.R-project.or). Primary R packages included “haven,” “survey,” “gtsummary,” and “ROCR.”

## 5 RESULTS

### 5.1 Participant characteristics

**Figure 1:**
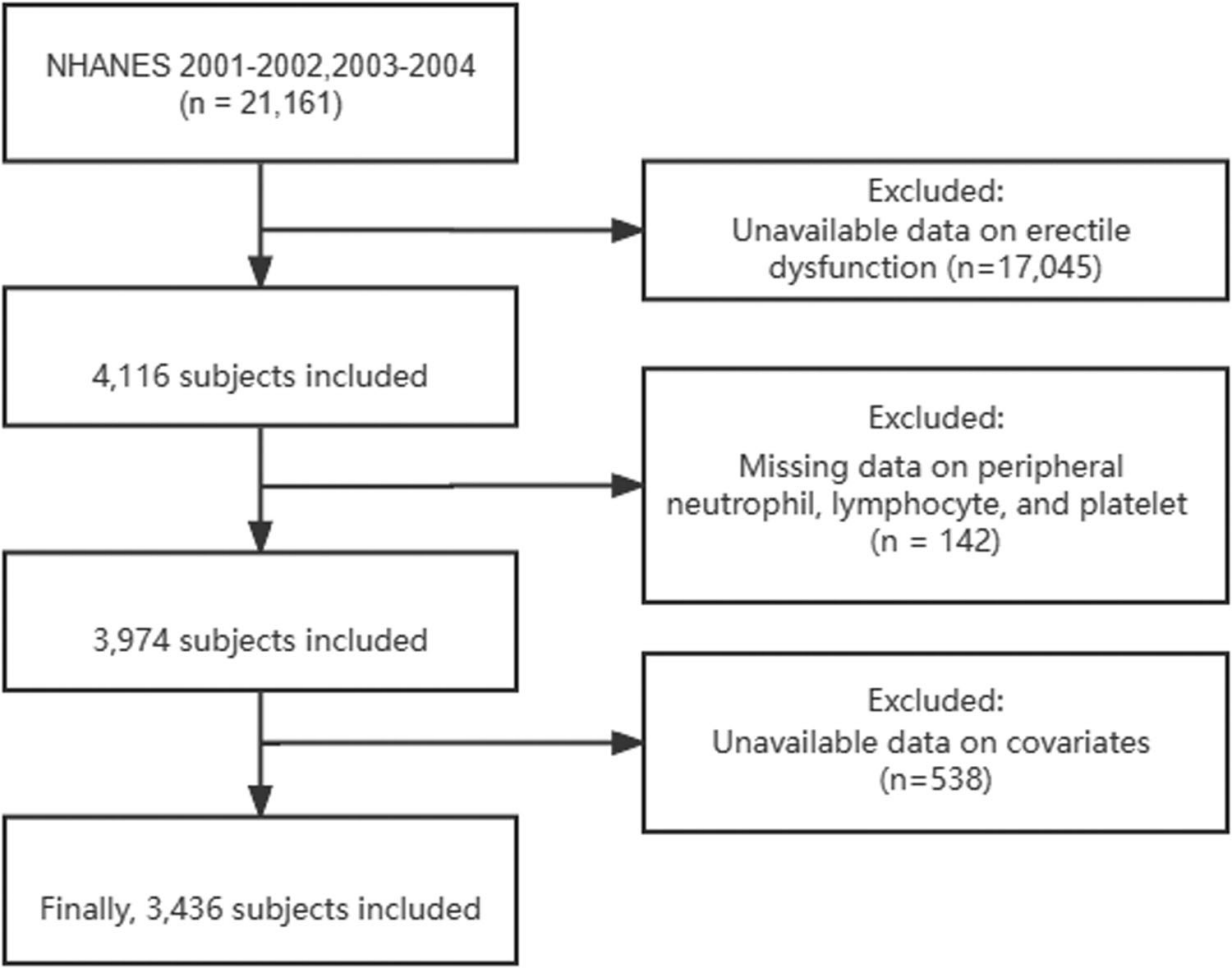
Flow chart selection process.

Initially, 21,161 participants obtained from the 2001−2004 NHANES data set. After application of the inclusion and exclusion criteria, 3436 participants were included in this study. The specific screening process was shown in Figure 1. Of these included participants, 916 described abnormal erectile function, while 2520 described normal erectile function. As shown in Table 1, in the ED group, approximately 80% of participants were ≥50 years old, 29% were less than high school, 19% were annual family income <$20,000, 35% were body mass index ≥30 kg/m^2^, 70% were smokes, 49% had hypertension, and 21% had cardiovascular disease. Notably, SII was statistically significant higher in participants with ED than in participants without ED (541.593 vs. 499.216, *p* = .007). Furthermore, the Q4 rate in ED group was significantly higher than that in non‐ED group (31% vs. 24%).

**Table 1:** Baseline characteristics of participants.

Characteristic	Erectile dysfunction		*p* a
	NO (*N* = 2520)	Yes (*N* = 916)	
Age (years)			<.001
<50	1700 (74%)	130 (20%)	
≥50	820 (26%)	786 (80%)	
*Race*			.3
Mexican American	498 (7.6%)	188 (6.8%)	
Other Hispanic	83 (4.0%)	33 (5.2%)	
Non‐Hispanic White	1364 (75%)	542 (77%)	
Non‐Hispanic Black	492 (9.5%)	136 (8.3%)	
Other Race—including Multi‐Racial	83 (4.1%)	17 (2.7%)	
*Marital status*			<.001
Married or living with partner	1723 (70%)	682 (78%)	
Living alone	797 (30%)	234 (22%)	
*Education level*			<.001
Less than high school	564 (13%)	367 (29%)	
High school or GED	660 (28%)	185 (23%)	
Above high school	1296 (59%)	364 (48%)	
*Annual family income*			<.001
Under $20,000	423 (12%)	255 (19%)	
Over $20,000	2097 (88%)	661 (81%)	
*Body mass index (kg/m^2^)*			.003
<25	770 (31%)	242 (25%)	
25−29.99	1053 (42%)	389 (40%)	
≥30	697 (28%)	285 (35%)	
*Alcohol intaking*			.010
Yes	2120 (85%)	741 (80%)	
No	400 (15%)	175 (20%)	
*Cigarette smoking*			<.001
Yes	1403 (55%)	652 (70%)	
No	1117 (45%)	264 (30%)	
*Physical activity status*			
Vigorous			<.001
Yes	1033 (45%)	160 (20%)	
No	1447 (54%)	680 (72%)	
Unable to do activity	40 (1.2%)	76 (7.8%)	
*Moderate*			<.001
Yes	1347 (59%)	396 (48%)	
No	1154 (40%)	472 (46%)	
Unable to do activity	19 (0.7%)	48 (5.5%)	
*Diabetes*			<.001
Yes	123 (3.4%)	218 (21%)	
No	2397 (97%)	698 (79%)	
*Hypertension*			<.001
Yes	586 (22%)	478 (49%)	
No	1934 (78%)	438 (51%)	
*Cardiovascular disease*			<.001
Yes	128 (4.0%)	217 (21%)	
No	2392 (96%)	699 (79%)	
SII (Continuous)	499.216 (363.237, 668.563)	541.693 (390.257, 750.284)	.007
*SII (Categorical)*			.009
Q1	730 (26%)	224 (22%)	
Q2	636 (25%)	202 (23%)	
Q3	597 (25%)	210 (25%)	
Q4	557 (24%)	280 (31%)	

Abbreviation: SII, systemic immune‐inflammatory index.

^a^ Chi‐squared test with Rao and Scott's second‐order correction; Wilcoxon rank‐sum test for complex survey samples.

### 5.2 The association between SII and ED

Weighted multivariable logistic regression was used to examine the relationship between SII and ED in each model, and found a significant connection between Q4 SII and increased ED risk. In model 3, the Q4 group had a 5% increased risk of ED compared with Q1 group. In model 1, Q4 group had the highest ED risk, which was 1.07 times higher than Q1 group.

**Figure 2:**
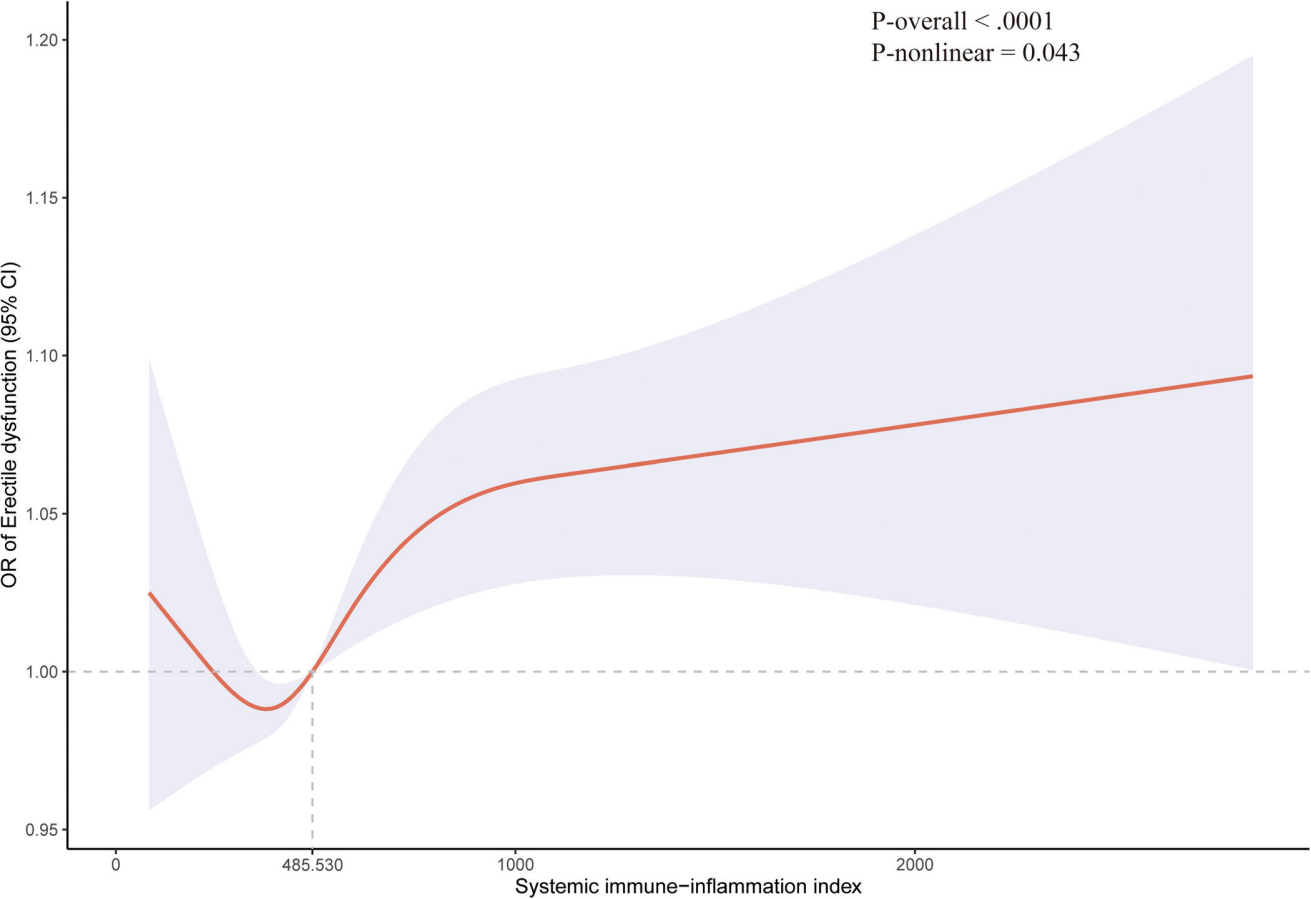
Restricted cubic spline for systematic immune‐inflammation index and erectile dysfunction.

RCS plot revealed a nonlinear association between SII and the risk of ED (Figure 2; *p*‐nonlinear = .043). Moreover, when the SII > 483.530, the risk of ED was significantly increased. As shown in Table 2, participants with SII > 483.530 had a higher ED risk than that of SII ≤ 483.530 in each model (odd ratio > 1, *p* < .05).

**Table 2:** Weighted multivariable logistic regression for the association between the SII and erectile dysfunction risk.

	OR (95% CI), P‐value		
	Model 1	Model 2	Model 3
*SII (Categorical)*			
Q1	Reference	Reference	Reference
Q2	1.01 (0.97, 1.05), 0.6	1.01 (0.98, 1.05), 0.5	1.01 (0.98, 1.05), 0.4
Q3	1.02 (0.98, 1.06), 0.3	1.03 (0.99, 1.07), 0.2	1.03 (0.99, 1.07), 0.13
Q4	1.07 (1.03, 1.11), 0.001	1.06 (1.02, 1.10), 0.005	1.05 (1.01, 1.09), 0.017
*SII (10^9^/L)*			
≤485.530	Reference	Reference	Reference
>485.530	1.04 (1.01, 1.07), 0.019	1.03 (1.01, 1.06), 0.018	1.03 (1.00, 1.05), 0.034

*Note*: Model 1: Unadjusted; Model 2: Adjusted for age, race, marital status, education level, annual family income, body mass index; Model 3: Adjusted for Model 2 + alcohol intaking, cigarette smoking, physical activity status, diabetes, hypertension, cardiovascular disease.

Abbreviations:95% CI, confidence interval; OR, odds ratio; Q1‐Q4, quartile 1‐quartile 4; SII, systemic immune‐inflammatory index.

### 5.3 Subgroup analysis

Subgroup analyses were performed to explore whether the SII‐ED association was steady in different stratifications. As shown in Table 3, participants with SII > 483.530 had a higher ED risk than that of SII ≤ 483.530 in age≥50, hypertensive, and non‐diabetes subgroup. However, no correlation was found in other subgroup. In addition, age and hypertensive may impact on the independent positive SII‐ED correlation (*p* value for interaction <.05).

**Table 3:** Subgroup analysis for the relationship between SII and erectile dysfunction risk.

	SII (10^9^/L)		*p*	*p* Value for interaction^a^
	≤485.530	>485.530		
*Age (years)*				.005
<50	Reference	1.00 (0.98, 1.02)	.7	
≥50	Reference	1.08 (1.03, 1.14)	.005	
*Body mass index (kg/m^2^)*				.643
<25	Reference	1.04 (0.99, 1.08)	.10	
25−29.99	Reference	1.03 (0.99, 1.07)	.2	
≥30	Reference	1.01 (0.97, 1.06)	.6	
*Hypertension*				.021
Yes	Reference	1.07 (1.03, 1.12)	.002	
No	Reference	1.01 (0.98, 1.04)	.5	
*Diabetes*				.438
Yes	Reference	1.09 (0.93, 1.26)	.3	
No	Reference	1.03 (1.00, 1.05)	.043	
Cardiovascular disease				.117
Yes	Reference	1.12 (1.00, 1.24)	.050	
No	Reference	1.02 (1.00, 1.05)	.10	

Abbreviation: SII, systemic immune‐inflammatory index.

^a^ Interaction analysis between selected subgroup and model 3.

### 5.4 The association between SII and severe ED

**Figure 3:**
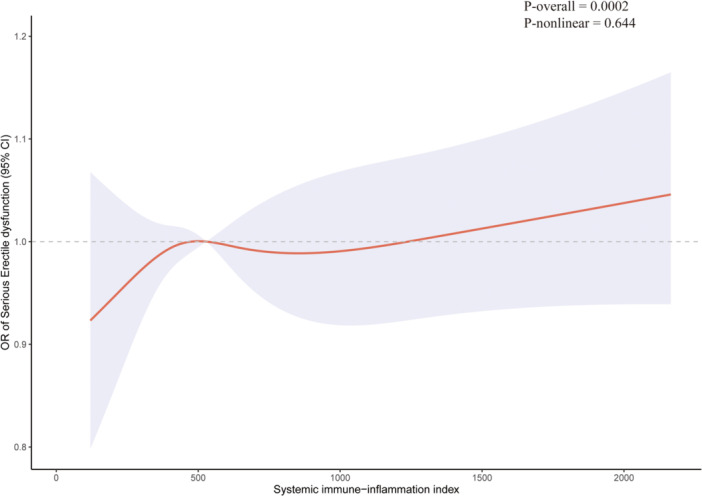
Restricted cubic spline for systematic immune‐inflammation index and severe erectile dysfunction.

Among the 916 ED participants, 345 had severe ED, while 571 had moderate ED. RCS used to explore the relationship between continuous SII and the risk of severe ED. The result found that the 95% CI cover 1, implying no relationship between SII and severe ED risk (Figure 3). Furthermore, weight multivariable logistic regression was used to evaluated the association between Q1 and Q4 SII and the risk of severe ED. As Table 4 shown, there was no association between categorical SII and severe ED risk. Hence, SII may not distinguish the severe ED (never able) and moderate ED (sometimes able).

**Table 4:** Weighted multivariable logistic regression for the association between the SII and severe erectile dysfunction risk.

	OR (95% CI), *p* Value		
	Model 1	Model 2	Model 3
*SII (Categorical)*			
Q1	Reference	Reference	Reference
Q2	1.01 (0.91, 1.13), 0.8	1.02 (0.92, 1.13), 0.7	1.00 (0.90, 1.13), >0.9
Q3	0.98 (0.89, 1.09), 0.7	0.99 (0.89, 1.10), 0.8	0.98 (0.89, 1.09), 0.7
Q4	1.00 (0.91, 1.09), >0.9	0.98 (0.90, 1.08), 0.7	0.97 (0.88, 1.07), 0.5

*Note*: Model 1: Unadjusted; Model 2: Adjusted for age, race, marital status, education level, annual family income, body mass index; Model 3: Adjusted for Model 2 + alcohol intaking, cigarette smoking, physical activity status, diabetes, hypertension, cardiovascular disease.

Abbreviations: 95% CI, confidence interval; OR, odds ratio; Q1−Q4, quartile 1‐quartile 4; SII, systemic immune‐inflammatory index.

## 6 DISCUSSION

In this cross‐sectional study, we have included 3436 participants of which 916 participants had ED. After the data analysis, we found an association between high level SII and the increased of ED risk. In a weight multivariable logistic regression that all covariate adjusted, the mean risk of ED for Q4 group was 1.05 times higher than Q1 group, and also higher than that of Q2 and Q3 group. For better clinical application, the RCS found that the risk of ED will increase significantly when SII is higher than 485.530. Similarly, weight multivariable logistic regression also supported the result that participants with SII > 485.530 had a 1.03 times higher ED risk than that of SII ≤ 485.530. Furthermore, subgroup analyses showed that SII was associated with the risk of ED in participants aged ≥50 years, hypertensive, and without diabetes. However, there was no significant relationship between SII and severe ED in further analyses. Therefore, SII may not be able to differentiate the degree of ED. Although SII has been proved to be associated with ED‐related disorders in other studies, our study was the first to analyze the relationship between SII and ED.

The SII is a novel inflammation‐related index based on platelet, neutrophil, and lymphocyte counts, which can reflect the balance between inflammatory response and immune response.^15^ Elevated SII can be explained by a relative increase in platelet count and the neutrophil count or a relative decrease in lymphocyte count.^11^ Platelets are recognized to be important mediators of system immune and thrombosis. The activated platelets produce chemokines and cytokines to enhance the activated recruitment of neutrophils. Meanwhile, a large number of inflammatory mediators will lead to the damage of vascular endothelial cell, and create a vicious cycle of platelet activation.^16^ Previous studies have found that adverse clinical outcomes were associated with platelet reactivity.^17^, ^18^ Neutrophils and lymphocytes are the subpopulations of leukocytes. Lymphocytes mediate adaptive immunity, and play a role in innate immunity. Neutrophils release proteolytic enzymes and reactive oxygen species that can kill microorganisms and aggravate inflammation. Therefore, platelets, neutrophils, and lymphocytes play vital roles in immune and inflammatory responses.

Nowadays, SII have been found to significantly elevate in metabolic, psychiatric, cardiovascular, inflammatory and oncological diseases. Mahemuti et al. performed a study based on the NHANES and found a nonlinear correlation between SII > 479.15 and increased the risk of hyperlipidemia.^10^ STING pathway activation and HDL‐related inflammatory may be potential mechanisms affected the association.^19^, ^20^ Zhu et al. performed a comparative study included 239 depressed patients and found that major depressed patient group had a higher SII level than the healthy group.^21^ Similarly, Wang et al performed a study based on the NHANES and found that high SII levels to be an independent risk factor for developing depression in diabetic patients.^13^ However, these study does not further explore the underlying mechanism. Akyüz et al. performed a single‐center study included 91 hypertensive patients and found that SII could be used as an independent predictor of non‐dipper hypertension.^22^ Moreover, SII > 578.25 was associated with a significantly increased risk of rheumatoid arthritis.^14^ In other male diseases, SII has been found to be associated with Peyronie's disease in men.^23^ Our study found an increased risk of ED in participants with SII > 485.530. Taking together the findings of above studies, our findings were reasonable.

ED is a complicated multi‐factor disease associated with various risk factors, including depression, diabetes, hypertension, dyslipidemia and cardiovascular disease. Previous studies have found a high levels of SII in depressed, hypertensive and dyslipidemia populations. However, no study has reported a correlation between SII and ED. Our study included 916 ED participants and found that ED group had a higher mean SII levels than the control group. Thus, our results support that inflammation have a role in the physiopathology of ED. Penile erection requires the normal function of endothelium, which product nitric oxide to increase arterial flow.^24^ Inflammation and oxidative stress can damage the normal vascular endothelium, leading to reduced NO production and restricted blood flow.^25^ Previous studies have found that other inflammatory indicators were also abnormally expressed in the ED patient. Liao et al. performed a comparative study included 113 ED and found that ED group had a higher neutrophil counts, neutrophil‐lymphocyte ratios, and platelet‐lymphocyte ratios, whereas lymphocyte counts were lower than those of control group.^26^ Similarly, Ventimiglia et al. performed a real‐life study included 279 ED patients and found that NLR > 3 may be an independent predictor of severe ED.^27^ Zhang et al. performed a meta‐analysis included 7 relevant studies and found that NLR and PLR can be independent risk factors for ED.^28^ In addition, serum CRP‐albumin ratio and serum high‐sensitivity CRP were also reported associated with ED risk.^9^, ^29^ PDE5i treatment can significantly reduce serum CRP levels. Hence, SII can be a valid reflection of ED risk.^9^ Previous studies have found that an elevated CRP/leukocyte ratio is associated with an increased risk of severe ED, suggesting an association between low‐grade inflammation and the degree of ED.^30^ However, the mechanism of this association needs to be clarified. Similarly, the reason why SII did not correlate with the degree of ED in the present study requires further investigation.

Some limitations are present in our study. First, our research is based on the NHANES database, which is limited to the US people. More research from other world regions is needed to confirm our conclusion. Second, the diagnosis of ED was from the self‐reporting of participants. Assessment based on the International Erectile Function Index questionnaire may better confirm ED patients. Third, some covariates that may influence ED, such as dyslipidemia and depression, were not collected in the research. Previous studies have reported that major depression can lead to ED development. Correcting for the interference of common confounders may better investigate the correlation between SII and ED risk.

## 7 CONCLUSION

In summary, our cross‐sectional study found a nonlinear correlation between SII and the risk of ED. Patients with SII > 485.530 had a significantly higher ED risk than those with SII ≤ 485.530. However, SII cannot distinguish severe ED and moderate ED. Further studies are needed to confirm our conclusion and explore potential mechanisms.

## AUTHOR CONTRIBUTIONS

**Di Chen and Quanhai LuoL**: Conceptualization and methodology. **Wenji Fan and Changsheng Chen**: Data acquisition. **Di Chen**: writing. Gang Liu: supervision. All authors approve the final manuscript.

## CONFLICT OF INTEREST STATEMENT

The author declare no conflict of interest.

## ETHICS STATEMENT

The studies involving human participants were reviewed and approved by the National Center for Health Statistics (NCHS) Research Ethics Review Committee. The patients/participants provided their written informed consent to participate in this study.

## Data Availability

All data are publicly available from the NHANES.
